# Optimal Descriptor Subset Search via Chemical Information and Target Activity-Guided Algorithm for Antimicrobial Peptide Prediction

**DOI:** 10.1021/acs.jcim.5c00600

**Published:** 2025-06-18

**Authors:** Luis A. García-González, Yovani Marrero-Ponce, César R. García-Jacas, Sergio A. Aguila Puentes

**Affiliations:** 1 Centro de Nanociencias y Nanotecnología, Universidad Nacional Autónoma de México, Km. 107 Carretera Tijuana-Ensenada, Ensenada, Baja California C. P. 22860, México; 2 Facultad de Ingeniería. Universidad Panamericana. Augusto Rodin No. 498, Insurgentes Mixcoac, Benito Juárez, Ciudad de México 03920, México; 3 Grupo de Medicina Molecular y Traslacional (MeM&T), Colegio de Ciencias de la Salud (COCSA), Escuela de Medicina, Edificio de Especialidades Médicas; Diego de Robles y vía Interoceánica, Universidad San Francisco de Quito (USFQ), Quito, Pichincha 170157, Ecuador; 4 Investigador por México, Secretaría de Ciencia, Humanidades, Tecnología e Innovación (Secihti), Ciudad de México 03940, México; 5 Tecnológico Nacional de México, Instituto Tecnológico de Mérida, Unidad de Posgrado e Investigación, Av. Tecnológico, Km. 4.5 S/N, Mérida 97000, México

## Abstract

Antimicrobial peptides (AMPs) have emerged as a promising alternative to conventional drugs due to their potential applications in combating multidrug-resistant pathogens. Various computational approaches have been developed for AMP prediction, ranging from shallow learning methods to advanced deep learning techniques. Additionally, the performance of shallow learning models based on self-learning features derived from protein language models has recently been studied. However, the performance of AMP models based on shallow learning strongly depends on the quality of descriptors derived via manual feature engineering, which may miss crucial information by assuming that the initial descriptor set fully captures relevant information. The AExOp-DCS algorithm was introduced as an automatic feature domain optimization method that identifies the “optimal” descriptor set driven by the chemical structure and biological activity of the compounds under study. QSAR models built on AExOp-DCS optimized descriptors outperform those using nonoptimized sets. In this study, we explore the use of AExOp-DCS to identify optimal descriptor subsets for AMP modeling. Experimental results show that the descriptors returned by AExOp-DCS contain information comparable to those used in top-performing models while exhibiting higher discriminative capacity. The generated models based on the descriptors returned by AExOp-DCS achieved performance metric values comparable to state-of-the-art approaches while utilizing fewer descriptors, suggesting a more efficient modeling process. By reducing dimensionality without sacrificing accuracy, this approach contributes to the development of more efficient computational pipelines for AMP discovery. Finally, a Java software called AExOp-DCS-SEQ is freely available, enabling researchers to leverage its capabilities for peptide descriptor search and AMP classification tasks.

## Graphical abstract

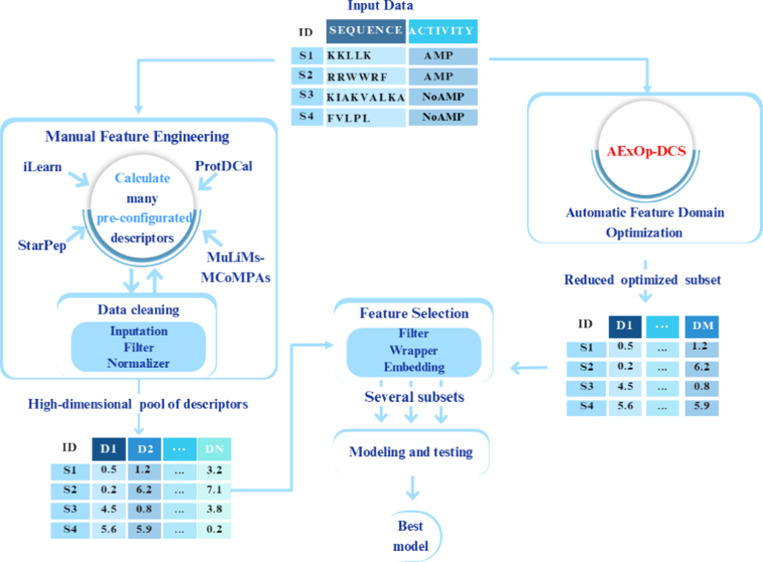

## 1 Introduction

Peptides are chains of amino acids consisting of up to 100 residues,^1^ where nearly 50% of their amino acids are hydrophobic^2^ and they possess a positive net charge ranging from +2 to +11.^2^ They play a crucial role in pharmacology due to their high specificity, affinity, and efficacy, as well as their low toxicity and immunogenicity.^3,4^ Additionally, they have diverse therapeutic applications, including antimicrobial, antitumor, or immunomodulatory activities.^1,3,4^ A particular interest has arisen in the development of new antimicrobial peptide (AMP)-based drugs due to their fundamental role in the immune system, where they act as direct inhibitors of pathogens, and modulators of both innate and adaptive immune responses.^5^ The discovery and development of AMPs have traditionally been time-consuming and expensive.^6^ Nonetheless, artificial intelligence (AI) and machine learning (ML) are revolutionizing this field by facilitating the efficient identification and design of novel AMPs.^1,3,4,6–8^

Shallow machine learning techniques are often employed for the identification and design of antimicrobial peptides (AMPs), especially when data availability is limited.^9^ The selection and extraction of molecular descriptors play a fundamental role in the construction of predictive shallow models for AMP prediction.^1,3,4,6,8,10–12^ In this context, handcrafted descriptors (HPDs) derived from physical and/or chemical properties have historically been the primary approach to codify relevant chemical information.^7,9,13–16^ In this sense, several algorithms have been developed to compute these descriptors.^17–21^ However, there is not a single method that can extract all the chemical information needed for different data sets. As a result, an initial high-dimensional pool of descriptors is generated.

This approach introduces two critical challenges. The first is to determine which descriptors should be considered. Each descriptor family codifies different aspects of the molecular structures.^22^ A traditional shallow-learning modeling pipeline addresses this challenge through a feature engineering process, as is shown in Figure 1. This usually involves computing a large set of predefined descriptors using tools such as iLearn^21^ or MuLiMS-MCoMPAS,^19^ followed by the execution of filtering techniques to discard irrelevant or redundant ones. These two steps are often repeated multiple times to refine the subset of descriptors used in the modeling process.

**Figure 1:**
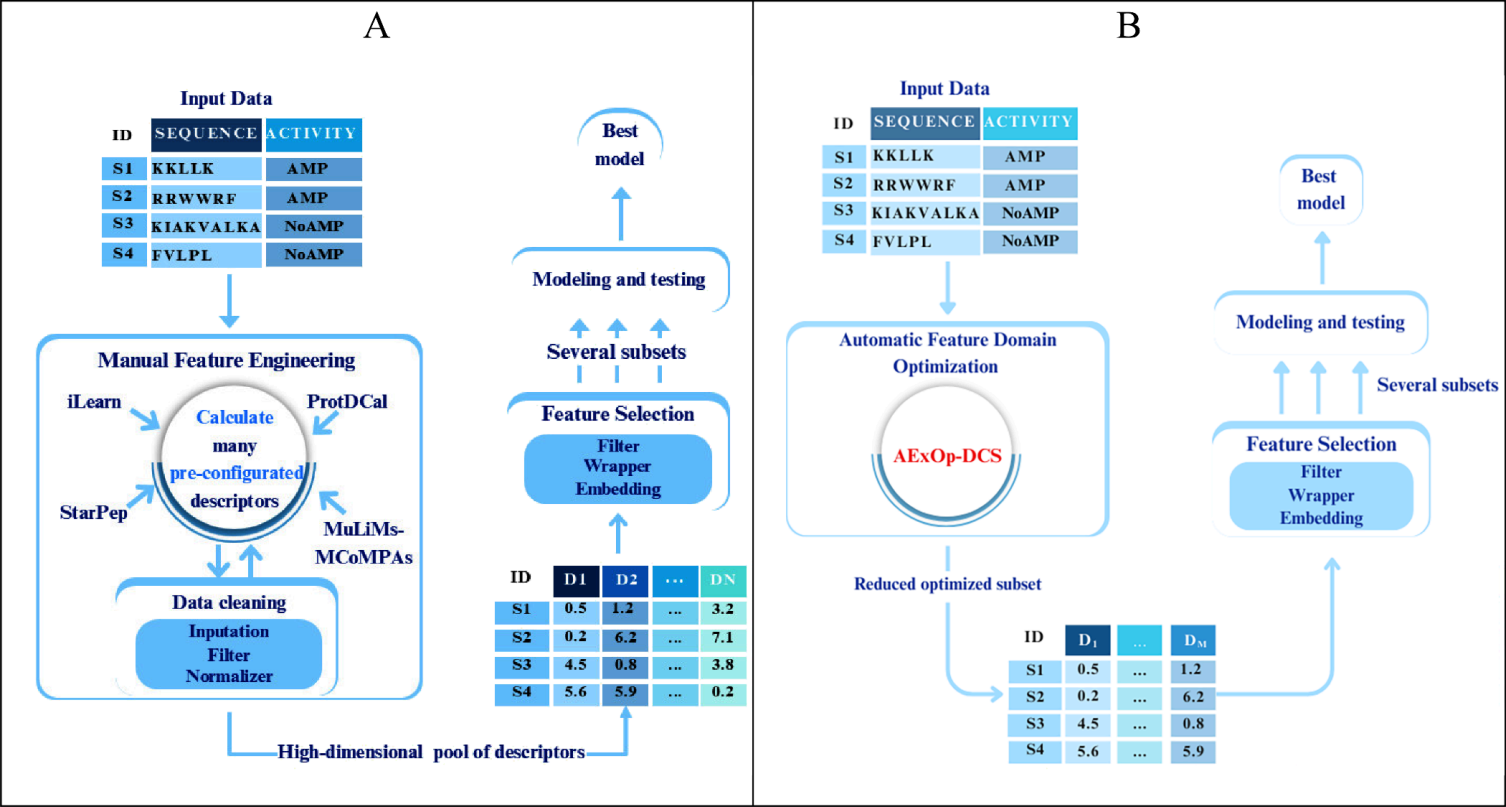
Optimized vs Traditional Workflows for AMP Classification. (A) Traditional workflow, where many descriptors are computed from peptide sequences before feature selection and modeling steps, (B) proposed workflow, where an optimized subset of descriptors is returned, followed by feature selection and model training.

The second challenge lies in selecting the most informative descriptors from the initial pool, a process that significantly influences the accuracy and generalizability of the models.^13^ Common feature selection methods include statistical tests, correlation analyses, recursive feature elimination, or embedded methods such as tree-based models.^7,9^ A good practice is to employ multiple feature selection techniques, enabling the construction of models from diverse, optimized subsets of descriptors. Additionally, feature fusion strategies may be used to combine the subsets obtained from different algorithms, thereby capturing complementary information and potentially enhancing model performance.^7^

The underlying assumption, which is not always fulfilled, is that the selected subset of descriptors will retain the highest predictive power and generalize well to unseen data. An effective feature selection process is essential to mitigate overfitting, reduce redundancy, and enhance both the interpretability of the final model and its generalization ability. However, this process is effort-demanding and computationally expensive. The initial descriptor pool often contains thousands of features, many of which may be irrelevant, and evaluating multiple combinations across different selection algorithms can significantly increase the computation time and resource consumption. Moreover, if the selection process is not carefully controlled, it may lead the exclusion of descriptors that are relevant but not individually strong. Another limitation stems from the descriptor generation tools themselves, which typically produce a fixed set of descriptors. This limits the ability to explore a broader space of possible descriptors within each family, potentially overlooking representations that could further improve model performance or interpretability.

On the other hand, a more conservative approach, such as starting with a small set of descriptors or using fewer feature selection techniques, may reduce computational cost but it likely does not analyse descriptors that are crucial for the specific modeling task. This trade-off between computational efficiency and descriptor comprehensiveness remains a major challenge in machine learning workflows. This challenge is no exception in the context of AMP prediction, where the effectiveness of models strongly depends on the selected descriptors.^6,8^ The ability of a model to accurately discriminate between active and inactive peptides relies heavily on how well the descriptors capture relevant sequence–function relationships. Therefore, it is essential to define a new approach that can autonomously generate a reduced relevant initial subset of descriptors. Such a strategy would help to prevent the unnecessary generation of features, reduce the time required for feature selection, and enhance overall model performance by starting from a more informative baseline.

To address these challenges, a new approach based on genetic algorithm, called AExOp-DCS,^13^ has been introduced. Unlike conventional approaches that generate many MDs and then select the most relevant ones, AExOp-DCS employs multicriteria evolutionary optimization to identify the best MDs according to the compounds and endpoints under analysis.^13^ As a result, a small subset comprising the most relevant descriptors found within the explored search space is obtained, enabling the application of the standard modeling pipeline over a lower dimensional subset, as shown in Figure 1.

AExOp-DCS determines this optimized descriptor subset through the exploration and fine-tuning of multiple descriptor configuration spaces (DCSs), where each DCS is defined as the set of parameters and their respective value domains used in a molecular descriptor calculation algorithm.^13^ For instance, the algorithm to calculate StarPep descriptors,^17^ takes as input an amino acid property­(*p*), a functional group­(*g*), a classical aggregation operator­(*c*), and a generalization invariant­(*i*).^17^ Thus, a DCS for the StarPep descriptor algorithm encompasses the possible values (domains) for each of its parameters, property (p), functional group (g), aggregation operator (c), and generalization invariant (i), as is shown in Figure 2. AExOp-DCS determines the best combinations of input parameters for each algorithm, with each DCS represented as a population of chromosomes, where each gene corresponds to a specific parameter in the MD calculation process.

**Figure 2:**
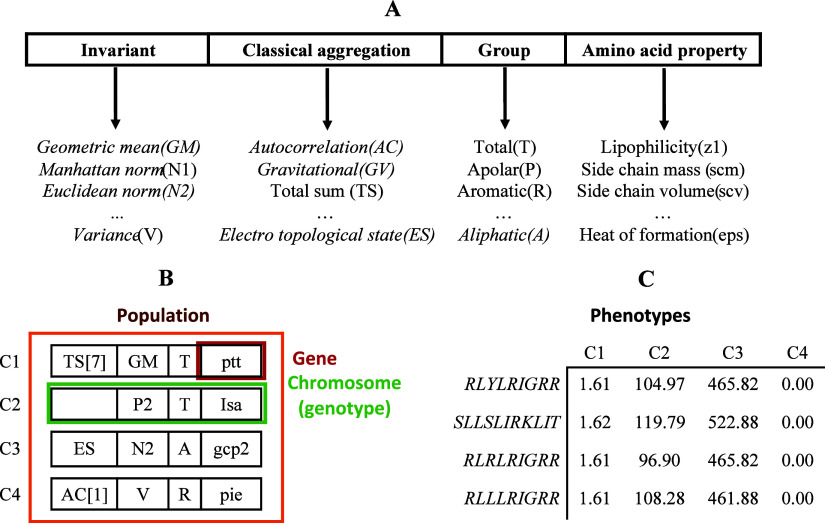
(A) Chromosome-based representation, (B) example of population, and (C) values (phenotypes) of the chromosomes of the population of example corresponding to the DCS of the StarPep algorithm.

Furthermore, the emergence of self-learned descriptors, such as those derived from protein language models like Evolutionary Scale Modeling models (ESM-2),^23^ offer an alternative to traditional HPDs by automatically capturing sequence patterns and functional relationships in a data-driven manner.^6–8^ These embeddings leverage architectures like transformers, recurrent neural networks, and convolutional neural networks allow to learn meaningful peptide representations without requiring explicit feature engineering.^4^ While their comprehensiveness is still being investigated, preliminary studies suggest that self-learned features and HPDs can complement each other in AMP prediction,^9,15^ paving the way for more efficient and scalable predictive models.

Therefore, the present work has three main objectives. First, it proposes the use of AExOp-DCS to search for an optimal subset of HPDs for AMP modeling. Second, it evaluates whether robust descriptors with high generalization ability can be obtained using AExOp-DCS. Finally, it analyses if combining HPDs with self-learned features leads to the development of more robust models.

To this end, the information content and the discriminative capacity of the descriptors returned by AExOp-DCS were analyzed using four state-of-the-art data sets.^7^ The returned descriptors were then compared with the ESM-2 embedding descriptors used to build the best models reported for each data set.^7^ Several models based on the descriptors returned by AExOp-DCS were built and assessed on these data sets to analyze their relevance in AMP prediction. Finally, several models were built based on the combination of ESM-2 embedding descriptors^7^ and the descriptors returned by AExOp-DCS to evaluate whether both types of descriptors are complementary and can enhance the predictive performance of the models.

## 2 Materials and Methods

### 2.1 AExOp-DCS to Search for an Optimum Peptide Descriptors Subset

AExOp-DCS is a genetic algorithm-based method designed to explore and optimize the DCS for multiple MDs.^13^ In a traditional genetic algorithm (GA), candidate solutions are encoded as fixed-length vectors or chromosomes, composed of parameters (genes), whose values (alleles) may belong to distinct domains. The algorithm generates an initial population with a fixed number of chromosomes, evaluates their fitness, and selects the most promising individuals (offspring) for recombination. Offspring may then be subjected to mutation before forming a new generation. This evolutionary process is repeated until a stopping criterion is met, and the chromosome with the highest fitness value is returned as the final solution.

Unlike traditional GA, the proposed algorithm does not start from a fixed set of precomputed descriptors. Instead, it explores the DCSs by evolving populations of parameter combinations. Each population corresponds to a specific DCS associated with a given MD calculation algorithm and each *n-length-chromosome* within a population represents a configuration of that algorithm. See Figure 2A for a definition of an n-length chromosome related to StarPep descriptors.^17^ Because there is no defined descriptor that fully codifies the chemical space of a peptide data set, AExOp-DCS returns a set of high-fitness chromosomes, each representing a descriptor configuration that best characterizes the chemical space under study.

As shown in Figure 2A, each gene encodes one of the *m* input parameters of the descriptor algorithm. For each population, a phenotype matrix (*F*) of size C*x*P is computed, where *C* is the number of chromosomes in the population and *P* is the number of peptides in the data set. The entry *F*_*i*, *j*_ represents the evaluation of the descriptor defined by the chromosome *i* in the peptide *j*. The chromosome fitness value is then computed based on the phenotype matrix. Figure 2B and Figure 2C show an example of this process using the StarPep descriptor algorithm.

AExOp-DCS starts the executions generating randomly a population for each DCS. For each population, the phenotype matrix is computed and a multicriteria fitness function is applied to determine the weight of each chromosome. This function considers the supervised metrics Relief-F,^24^ Pearson correlation, and the MDI index,^25^ along with the unsupervised Shannon entropy (SE)^26^ metric. The fitness for each chromosome is calculated then by aggregating the four calculated criteria through the Choquet integral.^27^

Once the fitness of each chromosome is computed, the genetic operators (selection, crossover, mutation and replacement) are applied to each population. Selection is performed via tournament selection, followed by a recombination with the Half Uniform Crossover (HUX)^28^ operator and a mutation process through scramble mutation.^29^ The new chromosomes replace their parents using a steady-state no-duplicate replacement strategy.^30^ Genetic operators are applied to each population independently, given that recombining configurations from two theoretically different MDs is not feasible.

Subsequently, the chromosomes in the new populations and the ones in current best subset found (initially empty) are merged into a total population. A Correlation Feature Subset (CFS)^31,32^ algorithm is applied to the entire population to obtain a subset with the best chromosomes. This subset will be the solution of the algorithm. A best-first strategy is used to guide the search of CFS. The algorithm runs for a predefined number of iterations, periodically restarting populations to explore new regions of the configuration space. The final output is an optimized set of descriptor configurations, enhancing molecular modeling and predictive tasks. See Algorithm 1 for additional details.

### 2.2 StarPep Peptide Descriptors

The algorithm for calculating protein descriptors using a weighting scheme based on amino acid properties and functional groups, known as StarPep,^17,33^ was considered to evaluate the effectiveness of AExOp-DCS in identifying the optimal peptide descriptor subset. This family of descriptors has been applied to explore the bioactive chemical space of AMPs through network-based approaches, clustering techniques, and similarity search models.^17,33^

The StarPep algorithm calculates peptide descriptors by applying different aggregation operators to a physicochemical amino acid property vector derived from a given peptide sequence (see Figure 3). This algorithm is based on four parameters. The first one is an amino acid property, which serves as a weighting scheme for each amino acid in the sequence. The second one is the chemical group to which the amino acid belongs to (e.g., polar). The third and fourth parameters corresponds to the aggregation operators to be applied to get the overall descriptor value.

**Figure 3:**
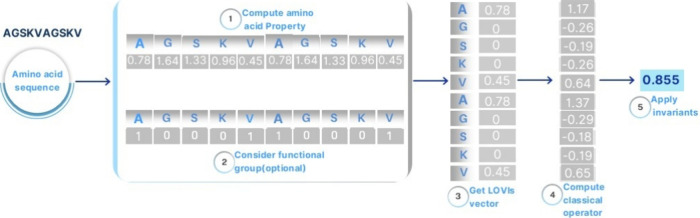
Workflow for computing a specific StarPep descriptor based on the Apolar group, relative frequency in reverse-turn (ptt) as the amino acid property, and electro-topological state and skewness as aggregation operators over the peptide *AGSKVAGSKV*. (1) Calculation of the property vector using ptt property. (2) Identify the amino acids in the apolar functional group (the entire sequence can be analyzed). (3) Compute the LOVIs vector by performing the Hadamard product between the property vector and the functional group vector. (4) Derive amino acid–based indices using the meta-descriptor electro topological state. (5) Apply skewness function as a generalization schema to obtain the peptide descriptor value.

As mentioned before, AExOp-DCS is based on the evolution of several DCSs.^13^ The DCS associated with descriptor StarPep is built from the four parameters on which it depends. As we consider 11 chemical groups, 20 amino acid properties, 137 non-classical aggregation operators and 24 classical aggregation operators, the DCS will have a dimension of 723360 StarPep descriptors.

### 2.3 Antimicrobial Peptide Data Sets

Four state-of-the-art data sets were used to ascertain if robust models can be built from the “optimal” sets of MDs returned by the AExOp-DCS algorithm. These data sets have been proposed to develop models to identify general-AMP (AMP),^34^ antibacterial (ABP),^35^ antifungal (AFP),^36^ and antiviral (AVP)^37^ peptide sequences. The four data sets considered were divided by their respective authors into training and test sets. The same partitions were used here to ensure comparability of results. Table 1 shows a description of the data sets, whereas a full description can be found in Section 2.1 of^7^.

**Table 1:** Training, Validation, and Test Sets of the Antimicrobial Peptide Datasets Used in This Work

	training data set			validation (tuning) data set			test data set		
data set	**total**	**positive**	**negative**	**total**	**positive**	**negative**	**total**	**positive**	**negative**
AMP	13430	6657	6773				7179	3530	3649
ABP	3120	1635	1485				9816	4017	5799
AFP	4124	2062	2062				2758	1379	1379
AVP	4908	2454	2454	1636	818	818	1636	818	818

## 3 Results and Discussion

The AExOp-DCS algorithm was applied to each training data set to obtain an ″optimal″ set of StarPep descriptors. The variability of each subset was analyzed using Shannon’s entropy (SE) to determine the information content of the descriptors returned by AExOp-DCS. Additionally, the predictive ability of these descriptors was evaluated using a single-variable model analysis. Next, several models were built to rate the performance of the descriptor subsets obtained from AExOp-DCS. The best models were then compared with those reported in the literature.^7^ Finally, several models were constructed by combining the subsets returned by AExOp-DCS with the ESM-2 descriptor subset. The latter has been used to build models reported in the literature as the best.^7^ This final step is aimed to evaluate whether both types of descriptors are complementary and able to enhance the predictive performance of the models. The quality of the generated models was measured using the Matthews correlation coefficient obtained via 10-fold cross-validation (*MCC*_10 – *cv*_), as well as the Matthews correlation coefficient on the test data sets *MCC*_*test*_. The specific settings used for the AExOp-DCS algorithm are described in Table SI1.

### 3.1 Analysis of the StarPep Descriptors Returned by AExOp-DCS

Four subsets comprised of **54, 123, 80** and **85** StarPep descriptors were returned by AExOp-DCS for the ABP, AFP, AMP and AVP training data sets, respectively. Over these subsets, a variability analysis was executed to verify if the returned descriptors contain high Shannon’s entropy (SE). SE measures the information content codified by descriptors, under the premise that descriptors with high entropy effectively discriminate the compounds in the data set.^14^ Additionally, the SE values of the returned descriptors and of the ESM-2 descriptors were compared.^7^ The SE values for both descriptor types on each training set are detailed in Table SI2.

For the ABP, AFP, AMP and AVP data sets, 65%, 70%, 79% and 84% of the StarPep descriptors exceed half of the maximum possible entropy. This suggests that the StarPep descriptors possess high information content, and thus they present good ability to discriminate between peptide sequences. Figure 4 compares the SE of the top 10 StarPep descriptors regarding to the top 10 ESM-2 descriptors. On the one hand, for the AFP and AVP sets, the AExOp-DCS algorithm returns StarPep descriptors with SE values greater than the ESM-2 descriptors. On the other hand, for the ABP and AMP sets, the StarPep descriptors with higher SE show similar SE values to those of the ESM-2 descriptors.

**Figure 4:**
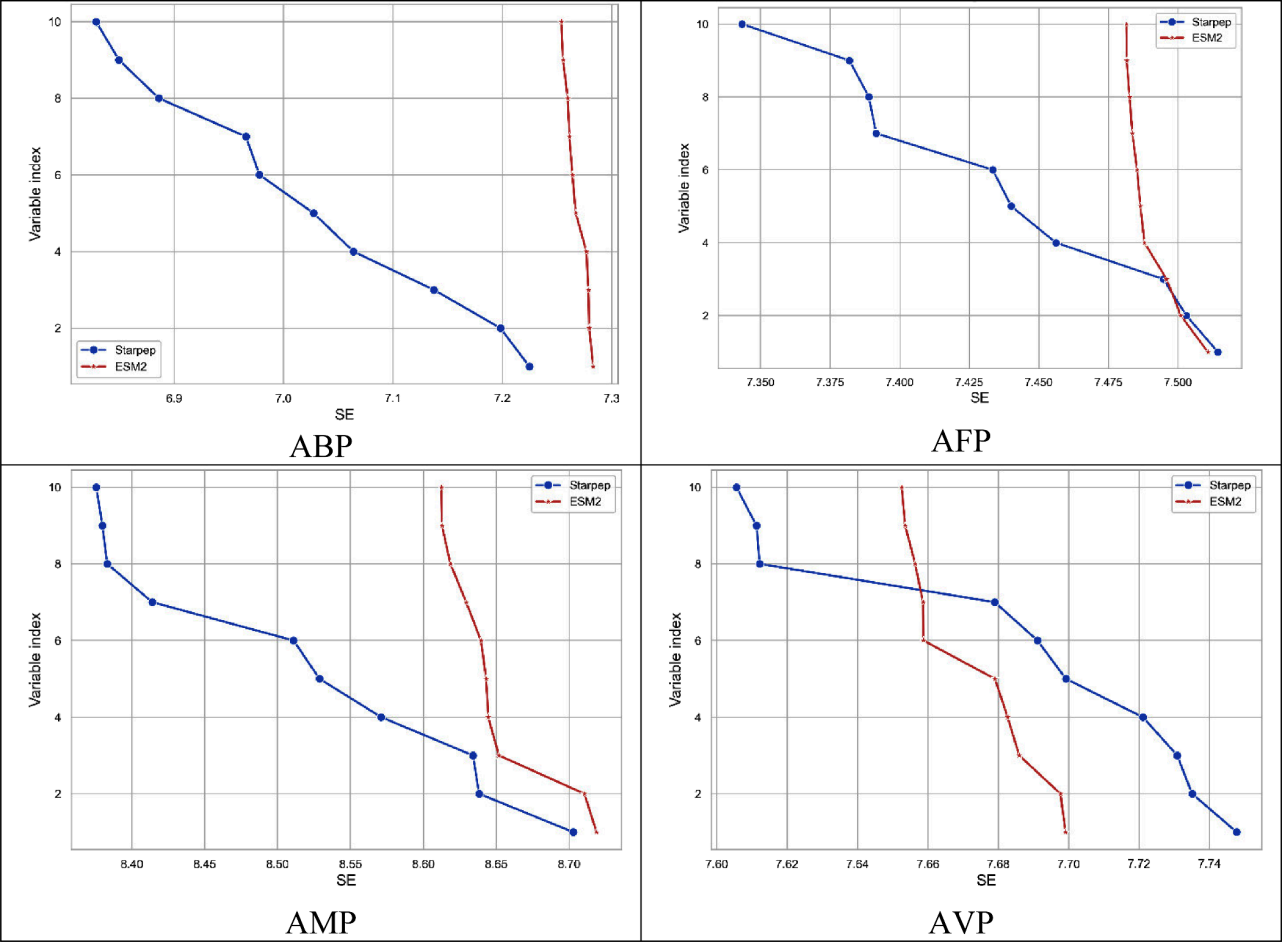
Comparison of the Shannon entropy distribution between the top ten StarPep descriptors returned by the AExOp-DCS algorithm and the top ten ESM-2 descriptors with the highest entropy for ABP, AFP, AMP, and AVP data sets.

Additionally, an univariate analysis of the predictive ability of the StarPep descriptors returned by AExOp-DCS was performed. The modeling methodology is described in Section SI1. This approach is typically used as an initial filtering step to discard descriptors with lower generalization ability. Subsequently, several one-descriptor models were built using the ESM-2 embedding descriptors.^7^ The performance of the best StarPep and ESM-2 one-descriptor models were compared using *MCC*_*train*_ values. Performance metrics for both types of descriptor are specified in Table SI3. Figure 5 presents boxplot graphics for each endpoint and descriptor type, showing the *MCC*_*train*_ values obtained by the best one-descriptor models. On the one hand, 321, 150, and 35 StarPep one-descriptor models achieved an *MCC*_*train*_ value above 0.5 for the ABP, AFP and AMP data sets, respectively. Specifically, for the ABP and AFP sets, 178 and 14 one-descriptor models outperformed all ESM-2 one-descriptor models. Also, it is important to highlight that for the ABP set, 14 one-descriptor StarPep models showed an *MCC*_*train*_ value superior to 0.8. On the other hand, for the AVP set, 101 StarPep one-descriptor models achieve *MCC*_*train*_ values greater than 0.4, where 49 of them outperformed the ESM-2 one-descriptor models.

**Figure 5:**
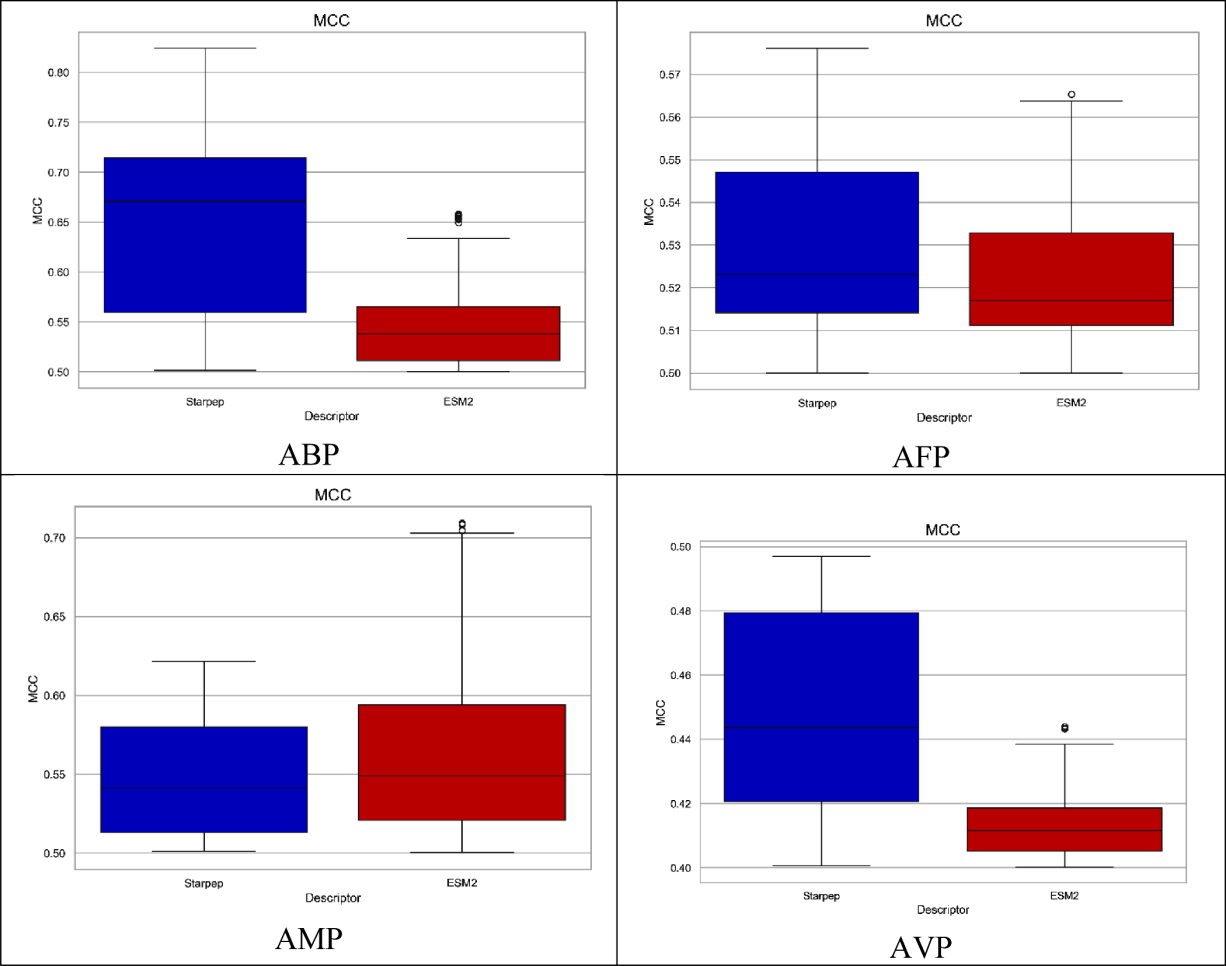
*MCC*_10 – *cv*_ values of the best one-descriptor models built from StarPep and ESM-2 descriptors for ABP, AFP, AMP, and AVP data sets.

Overall, StarPep descriptors contain similar information content but exhibit an univariate better discriminative capacity than ESM-2 descriptors. Moreover, StarPep descriptors are simpler and, leveraging the mathematical definition based on the aggregation of amino acid properties, they could be more easily interpreted. In contrast, ESM-2 descriptors are not easily interpretable due to their deep learning-based nature. Despite these differences, both descriptor types exhibit comparable information content, reinforcing the value of StarPep descriptors for a broad spectrum of discriminative studies.

### 3.2 Analysis of the Best StarPep and StarPep-ESM-models

In this section, we evaluate the generalization ability of the StarPep descriptors generated by AExOp-DCS. To this end, several models were built from the StarPep descriptors obtained for each data set. Additionally, to assess whether both descriptor types are complementary, several models were built by merging the StarPep descriptors and the ESM-2 descriptors (StarPep-ESM-2 models) that achieved the best performance in the univariate analysis.^7^

Model performance was evaluated on both training and test sets. For the training sets, accuracy (ACC), sensitivity (SEN), specificity (SPE), and MCC were calculated using a 10-fold cross-validation (10-CV). Generalization ability was assessed using the same metrics on the test sets. Additionally, for the AVP data set, the performance of the models in its calibration subset was reported. The *MCC*_10 – *cv*_ and *MCC*_*test*_ values were used for the comparisons. We analyzed models with *MCC*_10 – *cv*_ greater than 0.9 for the AMP and ABP data sets, as well as those with *MCC*_10 – *cv*_ greater than 0.8 and 0.75 for the AFP and AVP data sets, respectively. These thresholds were defined to guarantee comparability with the best models built for each data set.^7^ The performance metric values of the best StarPep and StarPep-ESM-2 models are described in Tables SI4 and SI5 respectively.

As a result, 14527, 1360, 25, and 1564 StarPep models with an *MCC*_10 – *cv*_ greater than the defined thresholds were obtained for the ABP, AFP, AMP and AVP sets, respectively. On average, StarPep models are comprised of 10, 20, 17, and 25 variables to predict the ABP, AFP, AMP and AVP sets, respectively. Figure 6 shows boxplot graphics related to the *MCC*_10 – *cv*_ and *MCC*_*test*_ values achieved by the best StarPep and StarPep-ESM-2 models. The *MCC*_10 – *cv*_ values achieved by StarPep models range from 0.900 to 0.959 for the ABP training set, 0.800 to 0.818 for the AFP training set, 0.900 to 0.903 for the AMP training set and 0.750 to 0.770 for the AVP training set. On the other hand, the *MCC*_*test*_ values range from 0.900 to 0.944 for the ABP test set, 0.800 to 0.826 for the AFP test set and 0.900 to 0.905 for the AMP test set. For the AVP sets, *MCC*_*tune*_ values range from 0.694 to 0.754 and from 0.702 to 0.765 for *MCC*_*test*_values.

**Figure 6:**
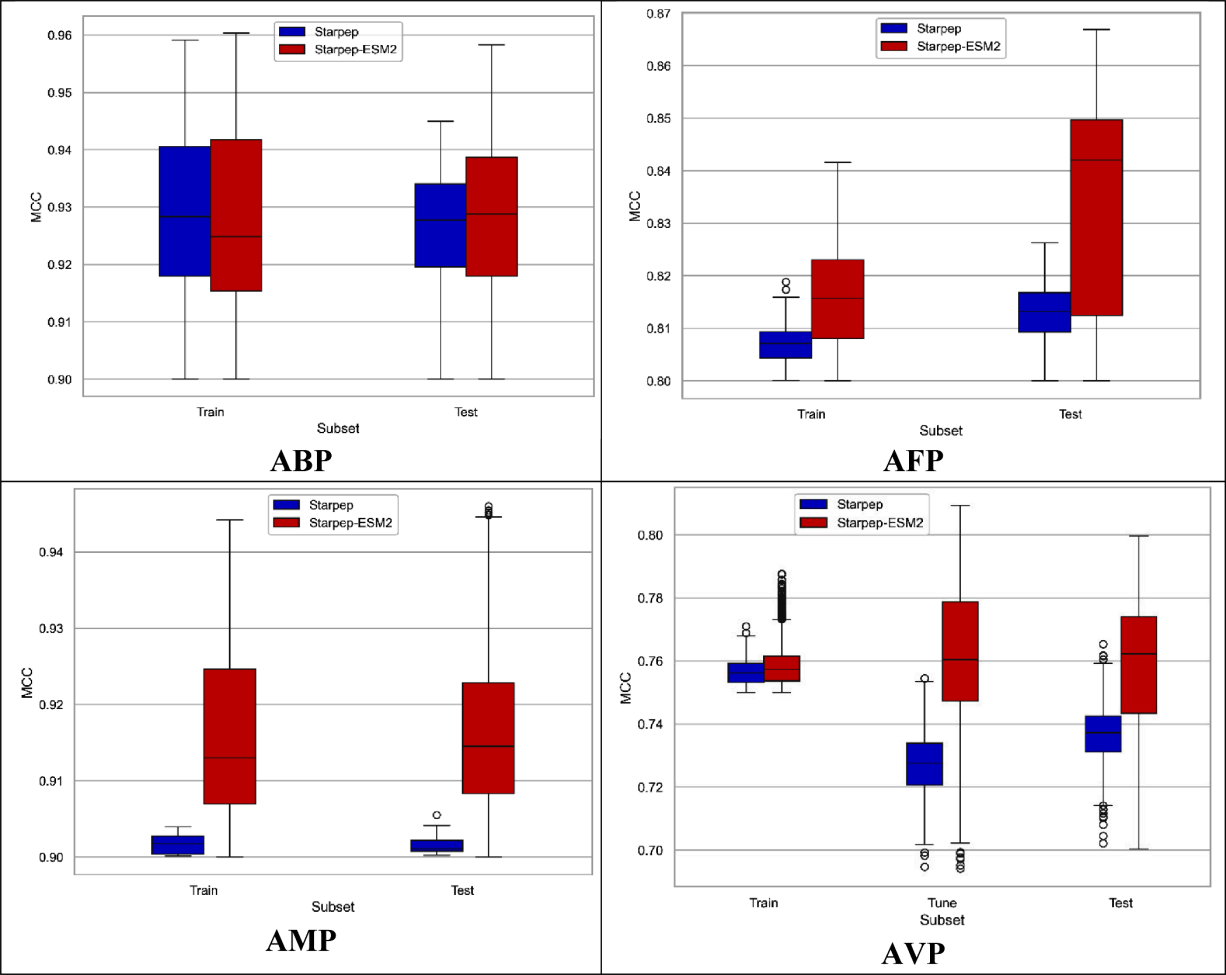
Boxplots related to the *MCC*_10 – *cv*_ and *MCC*_*test*_ values obtained in the train and test sets by the models built to predict ABP, AFP, AMP and AVP. In the case of AVP, it also *MCC*_*tune*_ values are shown.

Furthermore, 55408, 16560, 76372, and 5485 StarPep-ESM-2 models with an *MCC*_10 – *cv*_ greater than the defined thresholds were obtained for the ABP, AFP, AMP and AVP sets, respectively. On average, the StarPep-ESM-2 models are comprised of 11, 18, 21, and 22 variables to predict the ABP, AFP, AMP and AVP data sets, respectively. The *MCC*_10 – *cv*_ values achieved by StarPep-ESM-2 models range from 0.900 to 0.960 for the ABP training set, 0.800 to 0.841 for the AFP training set, 0.900 to 0.944 for the AMP training set, and 0.750 to 0.787 for the AVP training set. On the other hand, the *MCC*_*test*_ values range from 0.900 to 0.958 for the ABP test set, 0.800 to 0.867 for the AFP test set, and 0.900 to 0.946 for the AMP test set. For the AVP sets, *MCC*_*tune*_ values range from 0.694 to 0.809 and from 0.700 to 0.800 for *MCC*_*test*_ values.

In both cases, the StarPep and StarPep-ESM-2 models achieved *MCC*_*test*_ values greater than 0.9 for the ABP and AMP test sets, greater than 0.8 for the AFP test set and greater than 0.7 for the AVP test set. These results indicate that the models exhibit strong goodness-of-fit and generalization ability, suggesting that they are neither overfitting nor underfitting. Additionally, when comparing the *MCC*_10 – *cv*_ and *MCC*_*test*_ values between the best StarPep and the best StarPep-ESM-2 model, we can observe that *MCC*_10 – *cv*_ values for StarPep-ESM-2 models are higher than those for StarPep models in the AFP, AMP and AVP training sets by 2.3%, 4.1% and 1.7% respectively. Similarly, the StarPep-ESM-2 model outperforms StarPep models in *MCC*_*test*_ values by 4.1%, 4.1% and 3.5% in the AFP, AMP and AVP test sets, respectively. For the ABP data set, the difference is less than 1% for the train and test MCC values.

### 3.3 Comparative Analysis of the Best StarPep and StarPep-ESM-2 Models Built regarding the State-of-the-Art Models

Herein, we compare the performance of the best StarPep and StarPep-ESM-2 models with the best models reported in the literature. To this end, the top 50 StarPep and StarPep-ESM-2 models were selected and compared against the top 50 ESM-2 non-fused-feature models and the top 50 ESM-2 fused-feature models reported in the literature.^7^ The performance metric values of the top 50 StarPep and StarPep-ESM-2 models are described in SI6 and SI7, respectively. Table 2 shows *ACC*_10 – *cv*_, *MCC*_10 – *cv*_, *ACC*_*test*_ and *MCC*_*test*_ average values corresponding to top 50 StarPep, StarPep-ESM-2, ESM-2 nonfused-feature and ESM-2 fused-feature models. Additionally, the average number of features used by the best 50 models is given.

**Table 2:** Performances of the Top 50 StarPep Models, Top 50 StarPep-ESM-2 Models, and Top 50 State-of-the-Art Best Models^a^

end point	model	no. features	***ACC*_10*–cv*_**	***MCC*_10*–cv*_**	***ACC*_*tune*_**	***MCC*_*tune*_**	***ACC*_*ext*_**	***MCC*_*ext*_**
ABP	top 50 StarPep models	11(0.91)	0.98(0.004)	0.96(0.0009)			0.97(0.004)	0.94(0.0009)
	top 50 StarPep-ESM-2 fused-feature models	20(5.00)	0.98(0.003)	0.96(0.0006)			0.98(0.010)	0.95(0.002)
	top 50 ESM-2 nonfused-feature models [^7^]	80(31)		0.92(0.007)			0.94(0.003)	0.88(0.005)
	top 50 ESM-2 fused-feature models [^7^]	59(20)		0.92(0.008)			0.94(0.004)	0.89(0.007)
AFP	top 50 StarPep models	22(1.54)	0.91(0.010)	0.81(0.002)			0.91(0.012)	0.82(0.002)
	top 50 StarPep-ESM-2 fused-feature models	25(1.77)	0.91(0.007)	0.83(0.001)			0.93(0.021)	0.85(0.004)
	top 50 ESM-2 nonfused-feature models [^7^]	70(26)		0.83(0.009)			0.92(0.002)	0.84(0.003)
	top 50 ESM-2 fused-feature models [^7^]	77(19)		0.84(0.008)			0.92(0.002)	0.85(0.004)
AMP	top 50 StarPep models	17(1.51)	0.95(0.006)	0.90(0.001)			0.95(0.007)	0.90(0.001)
	top 50 StarPep-ESM-2 fused-feature models	33(1.02)	0.97(0.004)	0.94(0.001)			0.97(0.007)	0.93(0.001)
	top 50 ESM-2 nonfused-feature models [^7^]	109(31)		0.95(0.003)			0.97(0.001)	0.94(0.001)
	top 50 ESM-2 fused-feature models [^7^]	77(25)		0.94(0.003)			0.97(0.001)	0.94(0.002)
AVP	top 50 StarPep models	25(2.53)	0.88(0.021)	0.76(0.004)	0.87(0.026)	0.74(0.005)	0.88(0.015)	0.75(0.003)
	top 50 StarPep-ESM-2 fused-feature models	24(1.47)	0.89(0.014)	0.77(0.002)	0.88(0.056)	0.75(0.01)	0.89(0.021)	0.79(0.004)
	top 50 ESM-2 nonfused-feature models [^7^]	104(41)		0.78(0.012)			0.89(0.002)	0.78(0.004)
	top 50 ESM-2 fused-feature models [^7^]	102(24)		0.78(0.008)			0.90(0.002)	0.80(0.003)

a The standard deviation values are shown between parentheses.

Regarding the training sets, we can see as *MCC*_10 – *cv*_ values for StarPep and Startpep-ESM-2 models are, on average, similar between them and 4% higher than fused and nonfused feature models for the ABP training set. For the AFP training set, ESM-2 fused-feature models achieve the highest *MCC*_10 – *cv*_ value, being 1% and 3% greater than the StarPep-ESM-2 models and StarPep models, respectively. Moreover, ESM-2 nonfused-feature models present, on average, similar performance to the StarPep-ESM-2 models and a 2% better than the StarPep models. For the AMP training set, ESM-2 nonfused-feature models achieved the highest *MCC*_10 – *cv*_ value, being 1% and 5% better than StarPep-ESM-2 models and StarPep models, respectively. In contrast, ESM-2 fused-feature models perform, on average, similar to StarPep-ESM-2 models and 4% better than the StarPep models. As for the AVP training set, fused and nonfused-feature models have similar performance, achieving *MCC*_10 – *cv*_ values better than those the StarPep-ESM-2 and StarPep models by 1% and 2%, respectively.

On the other hand, regarding the generalization abilities, it can be observed that the StarPep-ESM-2 models, on average, achieved the highest *MCC*_*test*_ for ABP test set. In detail, StarPep-ESM-2 models outperform the ESM-2 fused and nonfused-feature models by 6% and 7% respectively. Additionally, StarPep models outperform the ESM-2 fused and nonfused-feature models by 5% and 6% respectively. For the AFP test set, StarPep-ESM-2 models and ESM-2 fused-feature models show the highest *MCC*_*test*_ values on average. StarPep-ESM-2 models are on average 1% better than ESM-2 nonfused-feature models. Regarding the AMP test set, fused and nonfused-feature models present similar performance, achieving *MCC*_*test*_ values better than those of the StarPep-ESM-2 and StarPep models by 1% and 4%, respectively. Finally, for the AVP test set, fused-feature models have the highest *MCC*_*test*_ values, followed by Starpe-ESM-2 models, ESM-2 nonfused-features models and StarPep models.

Additionally, Table 2 presents the average number of features used by each model across the data sets. We observed that, across all four data sets, StarPep and StarPep-ESM-2 models are built using the fewest features. For instance, in the ABP data set, StarPep models use only 1/7 of the features required by nonfused-feature models and 1/4 of the number of features used by the fused-feature models. Moreover, StarPep-ESM-2 models require only 1/4 and 1/2 of the features needed for nonfused and fused-feature models, respectively. Regarding the AFP and AVP data sets, StarPep and StarPep-ESM-2 models require three times fewer features than both ESM-2 fused and nonfused-feature models for the AFP data set. For the AVP data set, they require four times fewer features than both ESM-2 fused and nonfused-feature models. For the AMP data set, StarPep models require the fewest features, requiring only 1/2, 1/6 and 1/4 of the features used by StarPep-ESM-2, ESM-2 nonfused-feature and fused-feature models, respectively. In the case of StarPep-ESM-2 models, they required only 1/3 and 1/2 of the features used by ESM-2 nonfused-feature and fused-feature models, respectively.

In addition, Table 3 presents a comparison between the best models presented in this work and the best models of the state-of-the-art regarding the *ACC*_*ext*_, *Sen*_*ext*_, *SPE*_*ext*_ and, *MCC*_*ext*_ values. To this end, the top-1 StarPep, StarPep-ESM-2, ESM-2 nonfused-feature, and ESM-2 fused-feature models were considered for each data set. Additionally, the DL-based models AniAMPpred,^34^ Deep-ABPpred,^35^ Deep-AFPpred^36^ and Deep-AVPpred^37^ were considered. These models were proposed to predict AMP, ABP, AFP and AVP respectively. It can be analyzed in the Table 3 how, for each end point, StarPep and StarPep-ESM-2 are the models with the lowest number of features. For the ABP test set, it can be seen that the StarPep-ESM-2 models outperform the other models in the four metrics, followed by the StarPep model. Regarding the prediction of AFPs, the ESM-2 fused-feature model achieved the highest values for the *ACC*_*ext*_, and *MCC*_*ext*_ metrics, follows by the StarPep-ESM-2, ESM-2 nonfused-feature, Deep-AFPpred and StarPep models. For the AMP data set, both ESM-2-based models have the highest performance, followed by the StarPep-ESM-2, AniAMPpred, and StarPep models. Finally, for the AVP prediction, the AVPpred model outperforms the other models, followed by the ESM-2, StarPep-ESM-2 and StarPep models.

**Table 3:** Comparison between the Best Models Built in This Work and State-of-the-Art Modeling^a^

**end point**	**model**	**no. features**	***ACC*_*ext*_**	***Sen*_*ext*_**	***Spe*_*ext*_**	***MCC*_*ext*_**
ABP	StarPep	9	0.9717	0.9617	0.9861	0.9424
	StarPep-ESM-2	12	**0.9758**	**0.9677**	**0.9873**	**0.9505**
	ESM-2 nonfused-feature [^7^]	72	0.9491	0.9457	0.9514	0.895
	ESM-2 fused-feature [^7^]	72	0.9533	0.9435	0.9602	0.9035
	Deep-ABPpred [^35^]		0.958	0.96	0.9549	0.9138
AFP	StarPep	19	0.9108	0.934	0.8876	0.8225
	StarPep-ESM-2	23	0.9297	**0.9530**	0.9065	0.8603
	ESM-2 nonfused-feature [^7^]	64	0.9260	0.8970	**0.9550**	0.8535
	ESM-2 fused-feature [^7^]	93	**0.9315**	0.9181	0.9449	**0.8633**
	Deep-AFPpred [^36^]		0.9268	0.9159	0.9376	0.85
AMP	StarPep	14	0.9501	0.9515	0.9487	0.9002
	StarPep-ESM-2	30	0.9653	**0.9748**	0.9555	0.93074
	ESM-2 nonfused-feature [^7^]	147	**0.9741**	0.9669	0.9811	**0.9482**
	ESM-2 fused-feature [^7^]	82	0.9734	0.9646	0.9819	0.9469
	AniAMPpred [^34^]	200	0.9682	0.9499	**0.9860**	0.93
AVP	StarPep	24	0.8808	0.879	0.8826	0.7616
	StarPep-ESM-2	23	0.8973	0.8875	0.9071	0.7947
	ESM-2 nonfused-feature [^7^]	82	0.8973	0.8594	0.8973	0.7939
	ESM-2 fused-feature [^7^]	125	0.9028	0.89	0.9028	0.8059
	AVPpred [^37^]		**0.92**	**0.91**	**0.93**	**0.84**

a The highest value per test dataset is remarked in bold and underlined, whereas the second highest value is underlined only.

Therefore, the results highlight the quality of the StarPep descriptors returned by AExOp-DCS. First, their SE is similar to that of the ESM-2 descriptors, indicating that they capture a comparable content of information. Additionally, StarPep descriptors exhibit better generalization ability, enabling the construction of models with a well-balanced bias-variance trade-off. Furthermore, the StarPep models demonstrate competitive performance compared to the best models reported in the literature.

Thus, these results support the use of the AExOp-DCS to automatically determine good descriptor subsets, which demonstrated to be useful in discriminative tasks. In addition, with the use of this algorithm, it is avoided to carry out a traditional feature engineering process, which is mainly limited to use descriptors predefined in the different programs that are not necessarily the most suitable for the sequences and endpoints under analysis.

### 3.4 Software AExOp-DCS-SEQ

A Java-based software denominated AExOp-DCS-SEQ was developed to allow the search for an “optimal” subset of peptide descriptors based on the AExOp-DCS algorithm. AExOp-DCS-SEQ was developed using Java v11 ^38^ as language programming, IntelliJ IDEA 2024.3.1.1 as an integrated development environment (IDE)^39^ and Apache Maven v3.8.8 as build automation tool.^40^ Additionally, two in-house libraries starpep-md and aexop-api, were used. The library starpep-md (https://github.com/cicese-biocom/starpep-md) allows the calculation of the StartPep descriptors, while the library aexop-api (https://github.com/cicese-biocom/aexop-api) implements the logic related to the algorithm AExOp-DCS.

Currently, only StarPep descriptors are included in the software, but new descriptors can be easily added without modifying the logic of the algorithm. The code of AExOp-DCS-SEQ is available at https://github.com/cicese-biocom/aexop-dcs-seq. Additionally, the jar application can be found in the releases section. To execute the application is necessary to provide a fasta file with the sequences, a csv file with a column specifying the target value for each sequence in the fasta file and a project file that includes the configuration for algorithm execution.

## 4 Conclusions

This work proposes the use of the AExOp-DCS algorithm to search for an optimal subset of peptide descriptors. To this end, the performance of the StarPep descriptors returned by AExOp-DCS was evaluated on four peptide data sets. The results show that the descriptors returned by the AExOp-DCS algorithm have similar information content to the ESM-2 descriptors used for building the best models reported in the literature, along with a better discriminative capacity. Additionally, several models with comparable goodness-of-fit and generalization abilities were consistently created, but with a lower number of descriptors than several state-of-the-art models.

Therefore, it can be concluded that the AExOp-DCS algorithm is a suitable tool for determine relevant peptide descriptors, especially considering that StarPep descriptors are simpler. A Java application to search for an optimal subset of peptide descriptors was also released for free. Moving forward, we will continue the development of the AExOp-DCS-SEQ software, incorporating additional peptide descriptors and improving the performance of the AExOp-DCS algorithm. Furthermore, we will analyze the performance of the descriptors generated by the AExOp-DCS-SEQ software on other peptide data sets.

## Data Availability

The data supporting the reported results can be found at 10.6084/m9.figshare.28612025.v2.
